# The effect of a plasma next-generation sequencing test on antimicrobial management in immunocompetent and immunocompromised patients—A single-center retrospective study

**DOI:** 10.1017/ash.2022.356

**Published:** 2023-02-17

**Authors:** Denise Marie A. Francisco, Laila Woc-Colburn, Travis J. Carlson, Todd Lasco, Miriam Barrett, Mayar Al Mohajer

**Affiliations:** 1 Section of Infectious Diseases, Baylor College of Medicine, Houston, Texas; 2 Division of Infectious Diseases Emory University School of Medicine, Atlanta, Georgia; 3 Department of Clinical Sciences, High Point University Fred Wilson School of Pharmacy, High Point, North Carolina; 4 Department of Pathology and Clinical Microbiology, CHI St. Luke’s Health–Baylor St. Luke’s Medical Center, Houston, Texas

## Abstract

**Objective:**

To describe the use of next-generation sequencing (NGS) and to determine whether NGS leads to changes in antimicrobial management.

**Design and setting:**

This retrospective cohort study included patients aged ≥18 years admitted to a single tertiary-care center in Houston, Texas, with an NGS test performed between January 1, 2017, and December 31, 2018.

**Patients:**

In total, 167 NGS tests were performed. Most patients were of non-Hispanic ethnicity (n = 129), white (n = 106), and male (n = 116), with a mean age of 52 years (SD, 16). Moreover, 61 patients were immunocompromised: solid-organ transplant (n = 30), patients with human immunodeficiency virus (n = 14), and rheumatology patients on immunosuppressive therapy (n = 12).

**Results:**

Of the 167 NGS tests performed, 118 (71%) were positive. Test results associated with a change in antimicrobial management were found in 120 (72%) of 167 cases, with an average of 0.32 (SD, 1.57) fewer antimicrobials after the test. The largest change in antimicrobial management was in glycopeptide use (36 discontinuations) followed by antimycobacterial drug use (27 additions among 8 patients). Also, 49 patients had negative NGS results, but only 36 patients had their antibiotics discontinued.

**Conclusions:**

Plasma NGS testing is associated with a change in antimicrobial management in most cases. We observed a decrease in glycopeptide use after NGS results, which highlights physicians’ comfort in withdrawing methicillin-resistant *Staphylococcus aureus* (MRSA) coverage. In addition, antimycobacterial coverage increased, corresponding with early mycobacterial detection by NGS. Further studies are needed to determine effective ways to use NGS testing as an antimicrobial stewardship tool.

Analysis of microbiological culture data is a longstanding gold standard in diagnostics. Unfortunately, yield from cultures have been inconsistent and slow, prompting the need for faster and more sensitive methods. Metagenomic next-generation sequencing (NGS) is an ideal platform to meet this need because it is both sensitive and rapid. This technology relies on the sequencing of small fragments of circulating DNA in the cell-free component of the blood called cell-free DNA. Cell-free DNA has an estimated half-life in the circulation of <2 hours; hence, it has the potential to detect pathogens that are actively replicating.^1^One of these NGS tests is called the Karius test (Karius, Redwood City, CA); it can identify and quantify cell-free DNA in plasma from 1,250 clinically relevant bacteria, viruses, fungi, and eukaryotic parasites.^2^ The Karius test analytical sensitivity and specificity are >95% and >99%, respectively, and the clinical sensitivity and specificity are 93% and 63%, respectively.^2^ Plasma samples are collected from the patient and shipped to the Karius laboratory, where cell-free DNA are extracted and compared against a proprietary reference genome database to identify clinically relevant microbial DNA fragments in the plasma. A report containing a list of pathogens found in the plasma sample along with the quantitative concentration of the microbial cell-free DNA^2^ is sent to the ordering physician.

Plasma NGS tests are increasingly used as a diagnostic tool in infectious disease. Research is available regarding NGS tests aiding in the diagnosis of infections in human immunodeficiency virus (HIV) patients,^3^ allogeneic hematopoietic stem-cell transplant patients,^4^ sepsis,^2^ central nervous system (CNS) infections,^5^ cardiac infections,^6^ and respiratory^7^ infections. However, data regarding how NGS testing affects the antimicrobial prescribing practices of physicians are limited. A few retrospective cohort studies have been conducted to measure the clinical impact of plasma NGS testing, with differing outcomes and conclusions.^8–10^ Given the high sensitivity and corresponding low false-negative rate of the Karius test, we hypothesized that physicians would change their antimicrobial management following the receipt of NGS results, making it a possible antimicrobial stewardship tool. We sought to quantify the changes in antimicrobial management in both immunocompromised and immunocompetent patients who had an NGS test performed.

## Methods

### Study design

This retrospective cohort study was performed at a single tertiary-care center in Houston, Texas. All patients aged ≥18 years who had a NGS test performed between January 1, 2017, and December 31, 2018, were included. The study excluded patients whose NGS tests did not have a result due to an inadequate or inappropriate sample. These samples were rejected by the laboratory for the following reasons: <700 µL of plasma, plasma separated from whole blood >6 hours after the draw, plasma received at Karius at ambient temperature >4 days after draw, specimens have fewer than 2 patient identifiers, was collected in a tube after its expiration date or there was incomplete or improper separation of plasma. Some samples were rejected because they did not meet the internal quality control standards of the laboratory during testing.^11^ Most of the patients who had inadequate or inappropriate samples did have their plasma retested and their results were included in the study.

### Ethical considerations

The institutional review boards of Baylor College of Medicine and CHI St. Luke’s Health–Baylor St. Luke’s Medical Center (IRB study no. H-45143) approved this study.

### NGS test

In our facility, the use of NGS testing is limited to the list of active infectious diseases attending physicians, and although there is an active antimicrobial stewardship program, they did not have a separate role in approving the test or responding to the results.

**Fig. 1.**
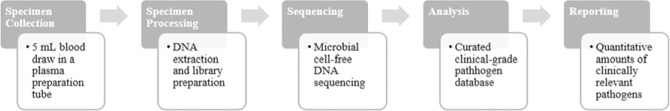
Figure showing the Next-Generation Sequencing Testing algorithm by the Karius laboratory.

The NGS test used in this study was the Karius test. Plasma was collected by hospital staff as part of routine clinical care and sent either fresh or frozen (if the sample is unlikely to reach the lab within 96 hours of the draw) to the Karius laboratory. All tests were performed by Karius based on their usual testing algorithm (Fig. 1).^2,11^

### Statistical analysis

Our primary outcome was change in antimicrobial management, defined as the number of antimicrobials discontinued or initiated at any point following the receipt of NGS results. Descriptive statistics were used to present demographics and to quantify changes in antimicrobial management. Unless otherwise noted, proportions are presented as percentages and continuous data are presented as means with range or SD. The Fisher exact test and the χ^2^ test were used to determine statistical significance along with ANOVA testing. SPSS version 24 software (IBM, Armonk, NY) was used for statistical analyses.

## Results

In total, 187 NGS tests were performed during the study period. Of those, 20 tests were excluded due to the inadequate samples, leaving 167 tests. Most of these patients were of non-Hispanic ethnicity (n = 129), white (n = 106), and male (n = 116), with a mean age of 52 years (SD, 16). Furthermore, 61 patients were immunocompromised: 30 were solid-organ transplant patients, 14 had HIV, 12 were rheumatology patients on immunosuppressive therapy, 1 patient was receiving hematopoietic stem-cell transplantation, and 1 had chronic granulomatous disease (Table 1).

**Table 1.** Patient Demographics and Comorbidities

Variable	Total N (%/range) N = 167	Next-Generation Sequencing Test Positive (%/range) N = 118	Next-Generation Sequencing Test Negative (%/range) N = 49
**Age (mean)**	52 (19-87)	51 (19-87)	54 (22-79)
**Gender**			
**Male**	116 (69%)	83 (70%)	33 (67%)
** Female**	51 (31%)	35 (30%)	16 (33%)
**Race**			
** Caucasian or White**	106 (63%)	75 (64%)	31 (63%)
** Black or African American**	38 (23%)	28 (24%)	10 (20%)
** Asian**	13 (8%)	9 (8%)	4 (8%)
** Others**	10 (6%)	6 (5%)	4 (8%)
**Ethnicity**			
** Hispanic**	36 (22%)	27 (23%)	9 (18%)
** Not Hispanic**	129 (77%)	89 (75%)	40 (82%)
** Unable to Determine**	2 (1%)	2 (2%)	0 (0%)
**Charlson’s Comorbidity Index (mean)**	3.70 (0-12)	3.74 (0-12)	3.61 (0-10)
**Immune System Status**			
**Immunocompetent**	106 (64%)	75 (64%)	31 (63%)
**Immunosuppressed**	61 (36%)	43 (36%)	18 (37%)
Human immunodeficiency virus positive patients	14 (23%)	13 (30%)	1 (6%)
Neutropenic^1^	3 (5%)	2 (5%)	1 (6%)
Solid Organ Transplant	30 (49%)	21 (49%)	9 (50%)
Hematopoietic Stem Cell Transplant	1 (2%)	0 (0%)	1 (6%)
Rheumatological	12 (20%)	7 (16%)	5 (28%)
Chronic Granulomatous Disease	1 (2%)	0 (0%)	1 (6%)

1 Neutropenia defined as absolute neutrophil count less than 1000.

An infectious diseases trained physician performed a retrospective review of the medical records of all patients to determine the indication for each NGS test. Each test was placed into 1 of 7 prespecified indication categories (Table 2). The most common indications were systemic or deep-seated infection in which a biopsy or other workup was negative or not possible (n = 50), fever of unknown etiology (n = 27), culture-negative endocarditis (n = 15), sepsis or shock of unknown etiology (n = 17), HIV patient with fever (n = 10), leukocytosis (white blood cell count >12 × 10^3^/mcL) of unknown etiology (n = 5), and transplant patient with fever (n = 5). The rest of the indications range from hyperbilirubinemia, rash, or encephalopathy of unknown etiologies to granulomas seen on biopsy with no infectious etiology detected on pathology (Supplementary Appendix).

**Table 2.** Indications for Next-Generation Sequencing Testing

Indications for Testing	Total N (%/SD)	Next-Generation Sequencing Test Positive (%/SD)	Next-Generation Sequencing Test Negative (%/SD)
	N = 167	N = 118	N = 49
Culture Negative Endocarditis	15 (9%)	10 (8%)	5 (10%)
Fever of Unknown Etiology	27 (16%)	21 (18%)	6 (12%)
Human immunodeficiency virus positive with Fever	10 (6%)	9 (8%)	1 (2%)
Transplant with Fever	5 (3%)	4 (3%)	1 (2%)
Systemic/Deep Seated Infection Where Biopsy or Other Workup is Negative or not possible	50 (30%)	35 (30%)	15 (31%)
Sepsis or shock of unknown Etiology	17 (10%)	13 (11%)	4 (8%)
Leukocytosis^1^ of unknown Etiology	5 (3%)	3 (3%)	2 (4%)
Others	38 (23%)	23 (19%)	15 (31%)

1 Leukocytosis defined as white blood cell count of greater than 12.00 x 10^3^/mcL

The average time from collection to the receipt of the sample at the Karius laboratory was 2 days (SD, 1), and the average time from collection to the receipt of the results from Karius was 3 days (SD, 1).

Of the 167 NGS tests performed, 118 (71%) were positive. Among the 118 positive results, 57 were gram-negative bacteria, 49 were viruses, 48 were gram-positive bacteria, 16 were fungi, 9 were atypical bacteria (5 *Bartonella henselae*, 2 *Rickettsia typhi*, 2 *Mycoplasma hominis*), 4 were mycobacteria (2 *Mycobacterium tuberculosis*, 1 *M. avium* complex, and 1 *M. neoarum*), and 4 were parasites (3 *Toxoplasma gondii* and 1 *Enterocytozoon bieneusi*). Also among these 118 positive NGS results, 50 had only 1 pathogen identified, but in 2 cases >10 pathogens were detected in the sample. Of the 68 tests with >1 pathogen identified, only 24 had multiple bacteria, whereas the remaining 44 tests identified coinfections with bacteria and other pathogens (ie, mycobacteria, viruses, fungi, and/or parasites).

NGS testing was performed after blood cultures were obtained in nearly all cases (Table 3). Of the 11 patients with positive blood-culture results, 7 matched with the plasma NGS results and 4 had discordant results. The discordant results were evenly spread between immunocompetent and immunocompromised patients, and these were coagulase-negative staphylococci or diphtheroids found on blood cultures that were not detected by the plasma NGS testing. Only 1 patient had a negative NGS result but a positive blood-culture result. Other non–blood cultures as well as serologic and molecular tests were performed for some patients (Table 3). Among 49 who had positive cultures or other serological testing results, 9 (17%) had negative NGS results.

**Table 3.** Other Diagnostic Tests Done with Plasma NGS Testing

Variable	N (%)	Statistics
**Blood Culture Testing**		
**Done**	148 (89%)	
Positive Blood Culture	11 (7%)	
Negative Blood Culture	137 (93%)	
**Not Done**	19 (8%)	
**Time NGS testing was done after Blood Culture (mean, SD)**	+ 3 days	SD = 3
**Other Diagnostics Tests**		
Culture based methods	93 (56%)	
Serological/PCR Tests	26 (16%)	
No Other Tests Done	48 (29%)	
**Other Diagnostic Test Results**		
Positive	50 (42%)	
Negative	69 (58%)	
**Time NGS testing was done after Other Diagnostic Tests Done (mean, SD)**	+ 5 days	SD = 7

**Fig. 2.**
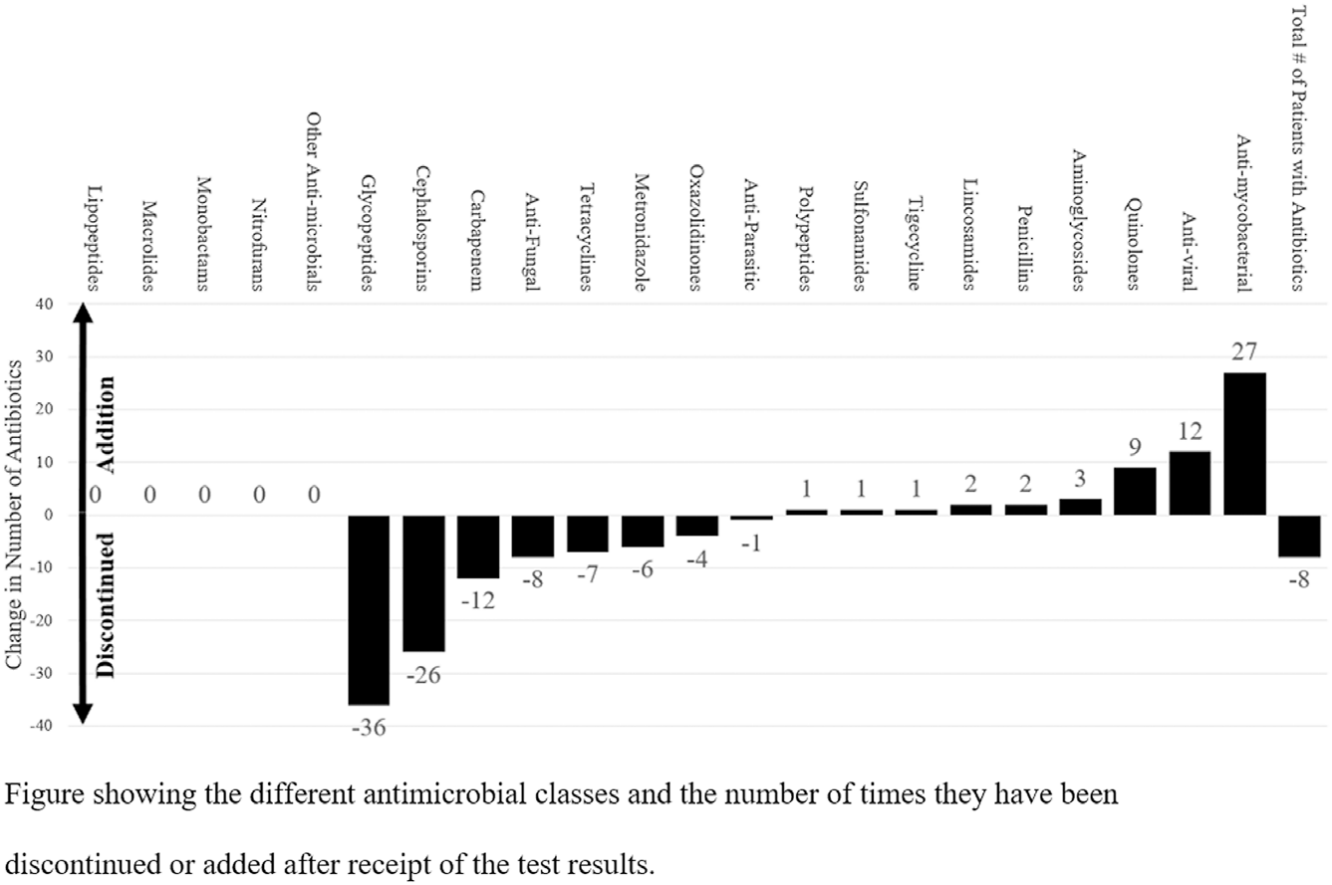
Change in Antimicrobial Management Following Next-Generation Sequencing Testing

In total, a change in antimicrobial management occurred within the first 24–72 hours in 120 patients (72%) following the receipt of NGS results. In those 120 patients, 158 antimicrobial changes were made: 100 antimicrobial discontinuations and 58 antimicrobial initiations. The largest change was found in glycopeptide use (36 discontinuations among 36 patients), and the next-largest change was for antimycobacterial drugs: 27 drug initiations among 8 patients (Fig. 2). Overall, the average number of antimicrobials per patient decreased from 2.42 (SD, 1.78) to 2.10 (SD, 1.84) following the receipt of NGS results, a change of −0.32 (SD, 1.57) antibiotics per patient. Lastly, although 49 NGS results were negative, only 36 of these patients had antimicrobials discontinued.

**Fig. 3.**
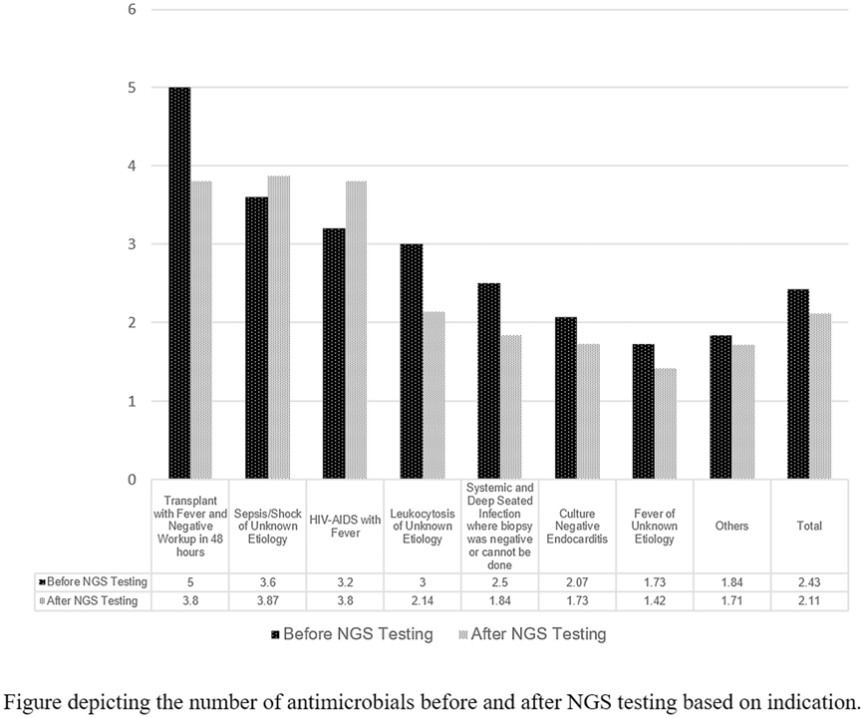
Change in Antimicrobial Management Following Next Generation Sequencing (NGS) Testing based on indications

A post hoc analysis was conducted to describe effect of NGS testing and the change in antimicrobial management based on indications. We did not detect a difference between positive and negative NGS tests between the 7 indications stated in Table 2, along with the miscellaneous other indications (Fisher exact test *P* = .566). We investigated the number of antibiotics before and after the NGS test (Fig. 3). If the test was used for transplant patients with fever, leukocytosis, or fever of unknown etiology, and systemic and deep-seated infection where biopsy was negative or could not be done, there was a net negative change in the number of antibiotics after the test. In the indication of HIV-AIDS with fever and sepsis or shock of unknown etiology, there was a net positive in the number of antibiotics used after the test. To further help determine which indications NGS testing may show the largest management adjustments, we calculated whether there was a statistical difference between the indications and the number of antibiotics changed. We did not detect a statistically significant difference based on analysis of variance (*P* = .148).

Further post hoc analysis was performed to describe plasma NGS results between the immunocompetent and immunocompromised. In total, 106 immunocompetent patients were tested: 75 were positive and 31 were negative. In the immunocompromised group, 61 patients had 43 positive results and 18 had negative tests. There was no statistically significant difference between the groups with regard to results (Fisher exact test, *P* = .97). When antibiotic changes were noted; in the immunocompetent group, 68% had antibiotic changes with a Δ (delta) change of −0.31 antibiotics after NGS testing. This result compares with the immunocompromised group, of whom 78% had antibiotic changes, with an average Δ of −0.33 antibiotics after the NGS test (Fisher exact test, *P* = .11).

## Discussion

Koch postulates^12^ have been the backbone of infectious diseases diagnoses for more than a century, but culture data can be slow and unreliable prompting the search for more rapid and specific diagnostic modalities that can improve patient outcomes. Plasma NGS testing, such as the Karius test, is an ideal platform to meet this need because it is both highly sensitive and rapid. In addition, the turnaround time is quicker than most culture-based testing. We observed an average time from sample collection to the receipt of the results at 3 days (SD, 1). The purpose of the present study was to quantify the changes in antimicrobial management in both immunocompromised and immunocompetent patients who had a NGS testing performed.

A few studies have addressed the clinical impact of NGS testing. A retrospective cohort study by Hogan et al^8^ was conducted across 5 US institutions in California where NGS testing was used. Clinical impact was based on standardized criteria ranging from positive (eg, there was a change in management or the NGS testing confirmed clinical diagnosis), negative (eg, NGS testing resulted in unnecessary treatment and diagnostic investigations and longer length of stay), none (eg, the NGS results were not acted upon) and indeterminate. Of 82 NGS tests performed, the results led to a positive impact in 6 cases (7%), negative impact in 3 cases (4%) and no impact in the remaining 87% of cases.^8^ This finding was echoed in a study by Wilson et al^9^ of NGS testing of cerebrospinal fluid (CSF). The study was a prospective multicenter case series conducted on both pediatric and adult patients at 8 US hospitals. In total, 58 central nervous system (CNS) infections were studied, and among them, CSF NGS testing identified 13 pathogens (22%) that were not identified by routine clinical testing at the hospital. Among these 58 infection cases, 45 cases (78%) had concurrent results between NGS and routine diagnostic tests. Among the 13 cases in which NGS testing helped with diagnoses, 8 had likely clinical effect and 7 guided treatment.^9^ Thus, in 86% of these cases, NGS testing had no clinical effect. Furthermore, the determination of “clinical effect” was subjective in both studies and was based on clinician judgement. In contrast, another study was done on pediatric patients with plasma NGS testing. In that study, cases were retrospectively classified by the authors as “clinically relevant” if there were management decisions based on the result. In that study, 56 (80%) of the positive NGS tests were deemed clinically relevant and 14 of those tests identified organisms where conventional testing modalities were not diagnostic.^10^ These discordant conclusions may be explained by the subjectivity of the study outcomes, along with the fact that while plasma NGS testing provides quantitative units in the final report, there are no clear threshold cutoffs that distinguishes colonization versus true infection.^8^ In our study, we aimed to measure the clinical utility of NGS testing in a more objective manner by quantitatively measuring changes in antimicrobial management following the receipt of NGS results.

In our study, most patients had conventional diagnostic testing performed prior to NGS testing. Additionally, NGS testing was done, on average, 3–5 days after conventional tests were performed. Many of these conventional tests results were negative. This finding contrasts with a previous study in which ∼33% of patients who had NGS testing performed already had a microbiological diagnosis prior to testing.^8^ Diagnostic practices of different clinicians vary regarding the use of NGS testing. In our study, 64% of NGS tests were performed on immunocompetent patients, whereas most other studies have focused on various immunocompromised populations. A post hoc analysis did not show any difference in our study between the immunocompromised versus the immunocompetent patients. The following questions remain: Should NGS testing be performed early during treatment as a rapid diagnostic tool similar to the SEP-SEQ trial?^2^ Or should NGS testing be performed when other conventional diagnostic tests have yielded negative results? Should NGS testing be limited to immunocompromised patients or used in immunocompetent patients too? Further research is needed to answer these questions.

We observed a modest reduction in the average number of antimicrobials per patient following the receipt of NGS results. The largest change involved the discontinuation of vancomycin. This finding highlights the clinicians’ relative comfort in stopping resistant gram-positive coverage if the NGS results did not show any bacterium that may necessitate glycopeptide use. Notably, our institution does not perform methicillin-resistant *Staphylococcus aureus* (MRSA) colonization screening. Although these findings are promising from an antimicrobial stewardship perspective, further studies, including cost-effectiveness studies, are needed to delineate the role of NGS testing in antimicrobial stewardship.

We also observed an increase in the prescription of antimycobacterial agents. Given the difficulty in diagnosing mycobacterial infections using conventional diagnostic tests, it is possible that plasma NGS testing could be used as a quicker diagnostic test for mycobacterial infections, a group of pathogens that are usually notoriously slow to grow and hard to culture. Although plasma NGS tests have a low clinical specificity, positive mycobacterial results are clinically relevant because mycobacterium in the plasma is commonly associated with a true infection rather than colonization. Interestingly, only 4 NGS tests were positive for mycobacteria, but 8 antimycobacterial antimicrobial initiations were identified. Although half may have been prompted by the NGS results, the other half were started for other reasons.

The strengths of this study include its larger sample size compared to other studies and objective primary outcome. A limitation of this study was its single-center nature; hence, the generalizability of these results is limited. Although we did analyze 167 NGS results, detection of statistically significant differences was limited by the small sample size and low power. Another limitation is the lack of secondary outcomes, including antimicrobial “appropriateness,” especially with the lack of antimicrobial sensitivity or resistance data in plasma NGS results for most detected pathogens. However, our goal was to use an objective measure of antimicrobial management. As previously mentioned, “clinical relevance” and “appropriateness” lack standardized definitions, which limits the external validity of other similar studies. Further studies and discussion on how to appropriately measure clinical relevance and appropriateness are needed to determine the true impact of NGS tests. Lastly, we were unable to determine whether each antimicrobial change was made solely based on the NGS results. In fact, that is likely not the case. Given the observational nature of this study and the medical complexity of these cases, we could not establish a causal relationship between NGS testing and antimicrobial management changes.

In conclusion, plasma NGS testing was associated with 158 antimicrobial changes (100 antimicrobial discontinuations and 58 antimicrobial initiations) among 120 immunocompetent and immunocompromised patients admitted to a single center in Houston, Texas. Specifically, glycopeptides were discontinued in 36 patients and antimycobacterial agents were started in 8 patients. An overall decrease in antimicrobial use was observed. Although 49 NGS results were negative, only 36 of these patients were taken off antimicrobials. Further studies are needed to determine the most effective ways to leverage NGS testing as an antimicrobial stewardship tool.

## Supplementary material

For supplementary material accompanying this paper visit http://doi.org/10.1017/ash.2022.356. click here to view supplementary material
