# Supplementary material for: The effect of a plasma next-generation sequencing test on antimicrobial management in immunocompetent and immunocompromised patients—A single-center retrospective study
Source: Antimicrob Steward Healthc Epidemiol. 2023 Feb 17;3(1):e31. doi: 10.1017/ash.2022.356 (PMC9972541; doi:10.1017/ash.2022.356)
Supplement: Supplementary file 1 [file S2732494X22003564sup001.docx]

**SUPPLEMENTARY APPENDIX:**

List of indications under the “Others” (n=38) tag:

- History of fever at home with lower extremity weakness - 1
- History of osteomyelitis, finished treatment, still with back pain - 1
- Abdominal aortic aneurysm leak with repair; intraoperative appearance concerning for infection and with a personal history of mycotic aneurysm who received prolonged antibiotics - 1
- Newly diagnosed HIV with lower extremity weakness; CSF studies negative - 1
- HIV patient with encephalopathy - 1
- HIV patient - 1
- Pre-solid organ transplant workup - 5
- Cough and shortness of breath in a solid organ transplant patient - 1
- Encephalopathy in a post solid organ transplant patient - 2
- Seizures in a post solid organ transplant patient - 1
- Diarrhea of unknown etiology - 1
- Fatigue of unknown etiology - 3
- Hyperbilirubinemia of unknown etiology - 2
- Muscle pain and rash of unknown etiology - 1
- Myocarditis, pharyngitis of unknown etiology - 1
- New lung infiltrates in a patient with extensive ID history - 1
- Lower extremity weakness with history of tick bite - 1
- Rash and altered mental status of unknown etiology - 1
- Sore throat and rash of unknown etiology - 1
- Septic arthritis - 1
- Pleural effusion - 1
- Granulomas seen on brain biopsy - 1
- Fungi seen on pleural biopsy - 1
- Anaerobes growing on pleural biopsy - 1
- Confirmation of known diagnosis - 5
- Determining when to stop Amphotericin B in a patient with known fusarium infection - 1
